# Using Propensity Score Matching Technique to Estimate Utilization and Costs of General Practitioners’ Services associated with Alzheimer’s Disease

**DOI:** 10.36469/9827

**Published:** 2016-03-28

**Authors:** Rajan Sharma, Elizaveta Sopina, Jan Sørensen

**Affiliations:** 1 Centre of Applied Health Economics Research; Unit of Health Promotion University of Southern Denmark, Odense, Denmark; University of Southern Denmark, Esbjerg, Denmark; 2 Centre of Applied Health Economics Research University of Southern Denmark, Odense, Denmark

**Keywords:** denmark, cost analysis, costs, propensity score, general practitioners, alzheimer’s disease

## Abstract

**Objective:** General practitioners (GPs) play an important role in caring for people with Alzheimer’s disease (AD). However, the cost and the extent of service utilization from GPs due to AD patients are difficult to assess. This study aimed to explore the principles of propensity score matching (PSM) technique to assess the additional GP service use and cost imposed by AD in persons aged ≥60 years in Denmark.

**Design:** PSM was used to estimate the additional use and cost of GP services attributable to AD. Case and control baseline characteristics were compared with and without the application of PSM. Propensity scores were then estimated using the generalized boosted model, a multivariate, nonparametric and automated algorithm technique.

**Setting:** Observational data from Statistics Denmark registry. Subjects: 3368 cases and 3368 controls; cases with AD were defined as patients with diagnoses G30 and F00 and/or those with primary care prescriptions for anti-AD drugs from the years 2004 until 2009. Main Outcome Measures: GP service utilisation and costs attributable to AD. Results: PSM brought a large improvement to the balance of observed covariates among the cases and control groups. AD patients received around 20% more GP services and utilized services that cost 15% more than non-AD controls during a calendar year. Conclusion: AD patients utilize more GP services and incur higher costs as compared to their matched controls. The PSM technique can be an effective tool to reduce imbalance of observable confounders from register based data and improve the estimations.

## BACKGROUND

Alzheimer's disease (AD) is a clinical syndrome caused by non-reversible neurodegeneration and is characterized by severe deterioration in cognitive ability.[1] The World Health Organization (WHO) has projected that AD and dementia will become the third-leading cause of burden of disease by 2030.[2] Around 15 000 new dementia cases are detected every year in Denmark.[3] The majority of dementia cases are AD patients. The prevalence of AD is projected to rise in the future with more elderly people and longer life expectancy. In Denmark, General Practitioners (GPs) are the primary caregivers for most chronic conditions. They play an important role in diagnostic evaluation and ongoing care as well as provide usual consultation services.[4] It is important to ascertain the difference in GP services use and cost between those with and without AD to assist with planning for future disease management. When the attributable cost related to dementia is estimated, it is important that the comparison is made for people with similar characteristics. That can be achieved by some form of matching technique. Propensity Score Matching (PSM) appears to offer a better match of observational covariates than simple sub-group matching. In comparison to other traditional regression methods, PSM has two major strengths. First, the matched cases and controls are only selected if they lie within the pre-assigned 'common support' region,[5] while regression methods have to rely on functional form of covariates. Secondly, PSM does not require functional form assumptions since it is non-parametric. Regression methods are based on certain assumptions such as linearity that might or might not hold true. Thus, the main aim of this research was to explore the use of PSM to estimate the impact of AD on service utilization and costs of GP services among patients with AD of ≥60 years of age.

## METHODS

### Subjects

The data for this study was made available by Statistics Denmark Research Services. Cases with AD were defined as inpatients and outpatients diagnosed with diseases having International Classification of Diseases (ICD)-10 codes G30 and/or F00 and those with primary care prescriptions for at least one of the drugs among donepezil, rivastigmine, galantamine and memantine from the year 2004 to 2009. The diagnostic data were obtained from the National Patient Registry (NPR)[6] and data related to drugs were obtained from the Danish National Prescription Registry (DNPR).[7] From these, a total of 3378 living AD cases were extracted.

The control group consisted of randomly selected 302 436 people in the general population for each year from 2004 to 2009. The selection of the control group was done by Statistics Denmark. All the cases and controls below the age of 60 were excluded from further study because of the low incidence of AD and for comparability. Thus, the propensity score estimation was based on 3378 cases and 302 436 controls which were alive by the beginning of 2011, and were residents for the entire year. On matching at the ratio of 1:1 within calipers, a total of 3368 AD cases and the same number of non-AD controls were matched resulting in a total sample of 6736. A total of 10 out of 3378 cases did not match with controls within the set criteria (that is within calipers = 0.25 * propensity score). A detailed illustration regarding subjects' selection for the study is shown in Table 1. Standardized mean difference and percentage improvement in the mean difference between treatment and control samples were used to quantify the performance of the generalized boosted model (GBM) and logistic models.

### Covariates

Covariates included marital status, region of residence, gender, age, highest completed education, comorbidity, hospital service utilization, early retirement status and taxable income. A dummy variable was created for living with a partner, including officially married (G), registered partners (R) and ambient living of two partners (L). The unmarried category also included divorced and widowed individuals. Similarly, a variable was created to recode the municipalities into five Danish regions. Education was grouped into seven categories: 'primary', 'secondary', 'bachelor', 'tertiary', 'long degrees', and 'PhD degree' and 'unknown' for missing data. In order to account for comorbidity, Charlson comorbidity scores were generated based on ICD-10 codes.[8] Charlson comorbidity index (CCI) scores were categorized into 3 levels: Low, moderate and high comorbidity, with Charlson comorbidity index (CCI) scores of 0-1, 2 and ≥3, respectively. Personal taxable income for the year 2010 was used for income variable. A small proportion of income data were missing in 2010 and were retrieved from the previous years. Similarly, a dummy variable was formed to include information on early retirement status.

**Table 1.** Study Flowchart

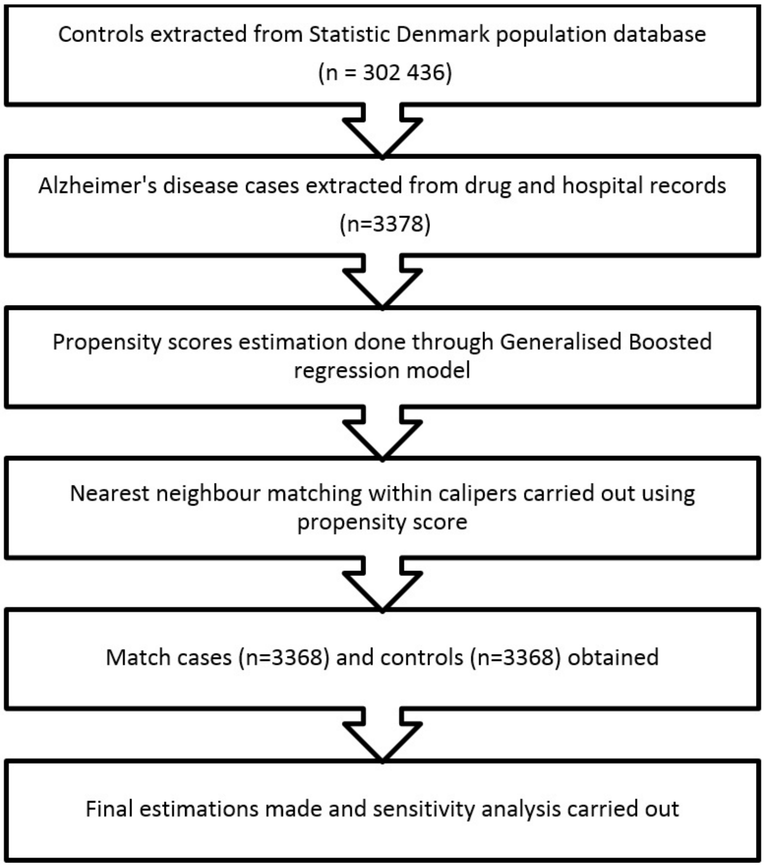

## Outcome Variables

Two dependent variables were used: the number of GP services utilized and gross payment made to GPs. Information about these variables was obtained from Danish health insurance data.

## Statistical Analyses

*Propensity Score Estimation: Generalized Boosted Model*

This study used 'twang' package in R software for GBM to estimate propensity scores. GBM is a data-adaptive, multivariate nonparametric and automated algorithm that fits several models through regression trees and merges the predictions of each model.[9] The number of trees assigned was 5000. The iterative fitting algorithm initiated with a single simple regression tree and a new tree was added at each new iteration, so that it provided the best fit to the model as compared to the previous iteration and increased the fit of the model for the data to create a perfect fit. The trees were indifferent to functional forms of covariates and provided the same propensity scores for any functional form of a variable specification. Four interaction depths were provided that determined the layers a tree can have. At this level of interaction depths, the software will consider two, three and four way possible interactions in the final model. A shrinkage factor of 0.01 was specified to reduce the impact of each additional tree in an effort to avoid over fitting.

*Matching: Nearest Neighbor Matching within Calipers*

This study used propensity scores obtained from GBM in order to match cases and controls to obtain matched dataset. 'Matchit' package in R implements matching through semi-parametric and non-parametric methods.[10] A caliper or an acceptable distance of 0.25 times the standard deviation of propensity score for each covariate was used in this study. 'Matchit' analyses are 'doubly robust' in the sense that inferences will be statistically consistent if either the matching analysis or the analysis models is correct.[10] The matching was done in such a way that the neighbourhood contains a control participant as a match for treated participant under the condition that absolute difference of propensity scores is the smallest among all possible pairs of propensity scores.

*Estimation*

After the PSM process was complete, Average Treatment Effect (ATE) and Average Treatment Effect on Treated (ATET) were estimated based on matched data using regression method in STATA 13.0. Estimations for service utilization and costs were carried out using Poisson regression and multiple linear regression, respectively. The concept of treatment effect is derived from Neyman-Rubin Framework.[9] Mathematically:

$$ATE(\tau) = E(Y_1 | z = 1) - E(Y_0 | z = 0) | X$$

Where $Y_1$ and $Y_0$ are the potential outcomes; $X$ = covariates included in the study and $Z=1$ and $Z=0$ denotes the presence or absence of AD, respectively. The '$E$' denotes the expected value. For the sample data, the estimation was done using the following equation:

$$\hat{\tau} = E(\widehat{y_1} | z = 1) - E(\widehat{y_0} | z = 0) | X$$

This assumes that selection of AD and non-AD cases depended on observable covariates $X$. Conditional on $X$, treatment assignment was assumed to be un-confounded $(y_0, y_1 \perp z) | X$.[11] Depending on underlying circumstances, difference in mean outcomes between two groups ($Z=1$ and $Z=0$) in the same condition is calculated.

Similarly, while estimating ATET, the interest was to find the difference between expected outcome values for patients with or without AD. Mathematical expression for estimating un-confounded ATET is given as:

$$\hat{\tau} = E(\widehat{y_1 - y_0}) | z = 1 | X$$

### Sensitivity Test

Rosenbaum sensitivity test for Hodges-Lehmann Point Estimate[12] was carried out to assess how robust the findings were to hidden bias due to unobserved confounder in this study. The 'hlsens' function within 'rbounds' package was used to carry out this test in R software.

## RESULTS

### Comparison of Performance of GBM and Logistic Methods in balancing Covariates

A standardized difference of less than 0.1 was considered to indicate a negligible difference in the mean of covariate between treatment groups.[13] The standardized mean differences in terms of propensity scores for all covariates were less than 0.1 in the case of GBM but not so in the case of the logistic method, as illustrated in Table 2. Moreover, even though percentage improvement in mean difference looked similar for both models, such improvements were larger in the GBM model.

### Baseline Characteristics before and after PSM

Table 3 shows the distribution of observable covariates in AD and non-AD groups. Imbalance in covariate distribution was greatly reduced by PSM. Estimations were, therefore, not impacted by the differences in observable confounding covariates. Apart from retirement status, none of the covariates have p-values less than 0.05 indicating a diminished difference between the groups. The mean age for both groups after PSM is almost identical (81 years). The mean difference of income was only around DKK (Danish Krone) 2900 in favour of non-AD subjects compared to DKK 21 000 before matching. Similar balances were also brought about in other covariates such as gender, marital status, educational status, regional distribution, retirement status, Charlson Comorbidity Index scores and hospitalization status.

### Service Utilization

As indicated in Table 4, ATE and ATET values for service utilization varied widely prior to PSM. This might be due to the different distribution of exogenous covariates in the case and control groups. ATET estimation for number of services used by people with AD was 3 (95% confidence interval [CI]: 2-5) from the mean of 34 (95% CI: 34-35) GP services. ATE for an average individual was 17 (95% CI: 14-20) in addition to the mean value of 28 services. PSM, ATE and ATET values almost coincided. On average, an AD patient was likely to use 7 (95% CI: 5-8) more services than the average of 31 (95% CI: 30-32) services in the population. This finding was highly statistically significant ($p<0.01$).

### Costs Related to GP Services

Table 4 illustrates the gross payments made (costs) for GP services. Prior to PSM, ATE and ATET values differed considerably. ATET value was DKK 97 more in addition to the mean of DKK 3639 in the controls (95% CI: 3608-3670). The ATE value prior to PSM was DKK 1976 (95% CI: 1553-2399) in addition to the mean of DKK 3143 (95% CI: 3131-3155). However, post-PSM, ATE and ATET values were similar. An average person with AD was likely to use services worth DKK 485 (95% CI: 296-676) more than the mean of around DKK 3250 (95% CI: 3129-3349) in the control group.

**Table 2.** Comparison of Performance in Balancing Covariates by Logistic and GBM Methods

| Variables | Propensity Score Estimation by Logistic Method | | Propensity Score Estimation by GBM[#] | |
|---|---|---|---|---|
| | **Standardized Mean Difference** | **% Improvement in Mean Difference** | **Standardized Mean Difference** | **% Improvement in Mean Difference** |
| **Male** | 1.570 | 94% | 0.011 | 96% |
| **Age*** | 4.342 | 97% | 0.000 | 100% |
| **Charlson Comorbidity Index Score Levels** | | | | |
| Moderate | 4.283 | 26% | 0.021 | 77% |
| High | 2.410 | 80% | 0.002 | 99% |
| **Married** | 6.832 | 73% | 0.025 | 90% |
| **Education** | | | | |
| Secondary | 1.403 | 95% | 0.002 | 99% |
| Bachelor | -3.650 | 8% | 0.000 | 100% |
| Tertiary | 0.000 | 100% | 0.041 | 60% |
| Long degrees | 0.969 | 92% | 0.021 | 84% |
| PhD and Research | 3.318 | 46% | 0.054 | 11% |
| Unknown | -11.966 | 55% | 0.019 | 93% |
| **Retirement Status** | 3.194 | 76% | 0.051 | 62% |
| **Regions** | | | | |
| Syddanmark | 4.0462 | 77% | 0.015 | 91% |
| Sjaelland | 0.9391 | 89% | 0.003 | 96% |
| Midtjylland | -2.020 | 81% | 0.009 | 91% |
| Nordjylland | -3.172 | 54% | 0.024 | 66% |
| **Hospitalization Status** | | | | |
| GPs Only | -4.578 | 88% | 0.024 | 94% |
| None | -8.131 | 77% | 0.029 | 92% |
| Income | -2.052 | 90% | 0.017 | 84% |

*Log of Age was used while estimating propensity scores by logistic regression.

[#] GBM is the model used for final estimations.

Propensity score estimation by logistic method was done using 'glm', a command for multiple logistic regression and balance statistics (standardized mean difference and percentage improvement in mean difference) were obtained using 'Matchbalance' command in R software with 500 bootstraps. Tertiary education includes Danish education level of 'Korte videregående uddannelser'; Long degrees include 'Øvrige mellemlange videregående uddannelser'.

GBM: General boosted model; GPs: General practitioners

**Table 3.** Baseline Covariates: Before and After PSM

| | Prior to PSM | | | | Post-PSM | | | |
|---|---|---|---|---|---|---|---|---|
| **Variables** | **Non-AD Controls (n=302 436)** | **AD Patients (n= 3378)** | **Total Sample (n= 305 814)** | **P-value** | **Non-AD Controls (n=3368)** | **AD Patients (n=3368)** | **Total Sample (n=6736)** | **P-value** |
| **Age** | 71 ± 8 | 82 ± 8 | 11* | 0.000 | 81.5 ± 0.137 | 81.5 ± 0.131 | 0.008* | 0.996 |
| **Gender** | | | | | | | | |
| Male | 139 259 (46.04%) | 1125 (33.3%) | 140 384 (45.91%) | 0.000 | 1103 (32.75%) | 1120 (32.25%) | 2223 (33.00%) | 0.660 |
| Female | 163 177 (53.96%) | 2253 (66.7%) | 165 430 (54.09%) | | 2265 (67.25%) | 2248 (66.75%) | 4513 (67.00%) | |
| **Education** | | | | | | | | |
| Primary | 121 962 (40.32%) | 1664 (49.26%) | 123 626 (40.43%) | 0.000 | 1642 (48.75%) | 1664 (49.41%) | 3306 (49.08%) | 0.871 |
| Secondary | 115 582 (38.22%) | 906 (26.82%) | 116 488 (38.09%) | | 900 (26.72%) | 903 (26.81%) | 1803 (26.77%) | |
| Tertiary | 56 242 (18.7%) | 433 (12.82%) | 56 675 (18.53%) | | 437 (12.98%) | 432 (12.83%) | 869 (12.90%) | |
| Not known | 8350 (2.76%) | 375 (11.10%) | 8725 (2.85%) | | 389 (11.55%) | 369 (11.25%) | 758 (11.25%) | |
| **Income (DKK)** | 203 703 ± 296 375 | 182 814 ± 98 586 | 20 889* | 0.000 | 185 740 ± 216 630 | 182 855 ± 98 519 | 2885* | 0.481 |
| **Regions** | | | | | | | | |
| Hovedstaden | 79 186 (26.18%) | 912 (27.00%) | 80 098 (26.19%) | 0.000 | 898 (26.66%) | 907 (26.93%) | 1805 (26.80%) | 0.870 |
| Syddanmark | 73 529 (24.31%) | 1096 (32.44%) | 74 625 (24.40%) | | 1070 (31.77%) | 1094 (32.48%) | 2164 (32.13%) | |
| Sjaelland | 51 205 (16.93%) | 472 (14.00%) | 51 677 (16.90%) | | 466 (13.84%) | 470 (13.95%) | 936 (13.90%) | |
| Midtjylland | 55 014 (18.19%) | 488 (14.44%) | 55 502 (18.15%) | | 499 (14.82%) | 488 (14.49%) | 987 (14.65%) | |
| Nordjylland | 43 502 (14.38%) | 410 (12.12%) | 43 912 (14.36%) | | 435 (12.92%) | 409 (12.14%) | 844 (12.53%) | |
| **Marital Status** | | | | | | | | |
| Unmarried | 83 211 (27.51%) | 1346 (39.85%) | 84 557 (27.65%) | 0.000 | 1384 (41.09%) | 1342 (39.85%) | 2726 (40.47%) | 0.297 |
| Married | 219 225 (72.48%) | 2032 (60.15%) | 221 257 (72.35%) | | 1984 (58.91%) | 2026 (60.15%) | 4010 (59.53%) | |
| **Early Retirement** | | | | | | | | |
| No | 288 362 (95.35%) | 3292 (97.45%) | 291 654 (95.37%) | 0.000 | 3309 (98.25%) | 3282 (97.45%) | 6591 (97.85%) | 0.023 |
| Yes | 14 074 (4.65%) | 86 (2.55%) | 14 160 (4.63%) | | 59 (1.75%) | 86 (2.55%) | 145 (2.15%) | |
| **Charlson Comorbidity Index Scores** | 0.44 ± 2.12 | 0.21 ± 1.08 | 0.2* | 0.000 | 0.24 ± 1.07 | 0.21 ± 1.08 | 0.02* | 0.347 |
| **Charlson Score Level** | | | | | | | | |
| Low | 275 584 (91.12%) | 3199 (94.70%) | 278 783 (91.16%) | 0.000 | 3200 (95.01%) | 3189 (94.69%) | 6389 (94.85%) | 0.684 |
| Moderate | 13 958 (4.62%) | 104 (3.08%) | 14 062 (4.60%) | | 92 (2.73%) | 104 (3.09%) | 196 (2.91%) | |
| High | 12 894 (4.26%) | 75 (2.22%) | 12 969 (4.24%) | | 76 (2.26%) | 75 (2.22%) | 151 (2.24%) | |

**Table 3.** Baseline Covariates: Before and After PSM (continued)

| Variables | Prior to PSM | | | | Post PSM | | | |
|---|---|---|---|---|---|---|---|---|
| | Non-AD Controls (n=302 436) | AD Patients (n=3378) | Total Sample (n=305 814) | P-value | Non-AD Controls (n=3368) | AD Patients (n=3368) | Total Sample (n=6736) | P-value |
| **Hospitalization status** | | | | | | | | |
| Only GP | 32 430 (10.72%) | 926 (27.41%) | 33 356 (10.91%) | 0.000 | 953 (28.3%) | 917 (27.23%) | 1870 (27.76%) | 0.330 |
| GPs & other PCPs | 260 763 (86.22%) | 2434 (72.05%) | 263 197 (86.06%) | | 2390 (70.96%) | 2433 (72.24%) | 4823 (71.60%) | |
| None | 9243 (3.06%) | 18 (0.54%) | 9261 (3.03%) | | 25 (0.74%) | 18 (0.53%) | 43 (0.64%) | |

*Mean difference; AD: Alzheimer's disease; GP: General Practice; PCPs: Primary Care Providers; PSM: propensity score matching

Continuous variables expressed as mean ± standard deviation; categorical variables expressed as N (%); The number of Alzheimer's patients (n=3378) is different from matched number of Alzheimer's and non-Alzheimer's patients, as 10 cases and controls did not match during nearest neighbor matching within calipers. PSM was done in two steps: First, estimation of propensity scores using generalized boosted regression. Second, nearest neighbor matching within calipers using propensity scores obtained in the first step.

Tertiary education in this table includes 'Bachelor', 'tertiary', ´long degrees' and 'PhD and research' levels.

**Table 4.** Annual (2011) GP Service Utilisation and Costs

| | | | GP Service Utilization | | | | | GP Service Cost (DKK) | | | | |
|---|---|---|---|---|---|---|---|---|---|---|---|---|
| | | | Coefficient | Robust SE | z | P>\|z\| | 95% CI | Coefficient | Robust SE | z | P>\|z\| | 95% CI |
| Prior to PSM (n=302 436 nonAD controls & n=3378 AD patients) | ATET | Additional services and costs for people with AD | 3 | 0.63 | 5.23 | 0.000 | [2-5] | 97 | 83.54 | 1.16 | 0.245 | [-67-261] |
| | ATET | Mean number and mean cost of services | 34 | 0.16 | 210.45 | 0.000 | [34-35] | 3639 | 15.92 | 228.58 | 0.000 | [3608-3670] |
| | ATE | Additional services and costs | 17 | 1.50 | 11.56 | 0.000 | [14-20] | 1976 | 215.70 | 9.16 | 0.000 | [1553-2399] |
| | ATE | Mean number and mean cost of services | 28 | 0.05 | 571.59 | 0.000 | [28-28] | 3143 | 6.19 | 507.29 | 0.000 | [3131-3155] |
| Post PSM (Final estimation) (n=2*3368) | ATET | Additional services and costs for people with AD | 7 | 0.76 | 8.73 | 0.000 | [5-8] | 484 | 98.307 | 4.92 | 0.000 | [291-676] |
| | ATET | Mean number and cost of services | 31 | 0.48 | 64.46 | 0.000 | [30-32] | 3257 | 58.272 | 55.90 | 0.000 | [3143-3372] |
| | ATE | Additional services and costs | 7 | 0.75 | 8.95 | 0.000 | [5-8] | 486 | 96.797 | 5.02 | 0.000 | [296-676] |
| | ATE | Mean number and mean cost of services | 31 | 0.46 | 66.84 | 0.000 | [30-32] | 3239 | 56.144 | 57.69 | 0.000 | [3129-3349] |

AD: Alzheimer's disease; ATE: Average Treatment Effect; ATET: Average Treatment Effect on Treated; CI: confidence interval; GP: General Practice

### Sensitivity analysis

Sensitivity analysis was carried out after separate matching process in R using 'Matching' package using logit model to estimate propensity scores along with 500 bootstraps which performed poorly as compared to GBM (Table 2). The maximum value of Gamma (Γ) was set to be 2 with increments of 0.1. In the absence of hidden bias, the median difference in gross payment made to GPs was only DKK 30.5. It is important to note that the median difference was smaller than the mean difference. Table 5 demonstrates that once the gamma value increased by 0.1, the bounds denoting the significance level included zero as the lower bound turned negative. This suggests the GP gross payment estimate was not robust and the finding was sensitive to possible hidden bias due to an unobserved confounder. In the sensitivity test for the number of services received from GPs, the lower bound turned negative only when the gamma value increased to 1.3. The median difference in number of services received in absence of hidden bias was 4.

**Table 5.** Sensitivity Analysis

| Gamma Values | GP Service Utilization | | GP Service Costs (DKK) | |
|---|---|---|---|---|
| | Lower bound | Upper bound | Lower bound | Upper bound |
| 1.0 | 4.0* | 4.0* | 30.5* | 30.5* |
| 1.1 | 2.5 | 5.1 | -100.1 | 162.0 |
| 1.2 | 1.0 | 6.1 | -219.6 | 282.0 |
| 1.3 | -3.7e | -05 7.6 | -329.1 | 393.0 |
| 1.4 | -1.0 | 8.6 | -431.1 | 496.0 |
| 1.5 | -2 | 9.6 | -526.1 | 592.5 |
| 1.6 | -3 | 10.6 | -615.1 | 684.5 |
| 1.7 | -3.5 | 11.1 | -699.6 | 771.5 |
| 1.8 | -4.5 | 12.1 | -779.1 | 853.5 |
| 1.9 | -5 | 13.1 | -854.6 | 931.5 |
| 2.0 | -6 | 13.6 | -927.1 | 1006.5 |

*Unconfounded estimate from Rosenbaum Sensitivity Test for Hodges-Lehmann Point Estimate
Gamma is log odds of differential assignment to treatment due to unobserved factors
DKK: Danish Krone; GP: General practitioner

### Discussion

This study used PSM technique that has a number of advantages over traditional regressions[14-16] and experimental evaluation techniques.[17] First, it avoids ethical considerations that occur in Randomized Controlled Trials when both treated and control groups do not receive equally effective treatments. Second, data generation is usually less costly as already available data can be used. Third, possibilities regarding the loss of treatment and control patients are less as compared to randomized assignment. Compared to experimental techniques, PSM has certain limitations. First, PSM can only take into consideration the observable characteristics, as it requires conditional independence assumption, while in other experimental techniques with randomization the treated and non-treated populations are similar for both observed and non-observed characteristics. Second, experimental techniques ensure common support across the whole sample through random assignment, while PSM can only estimate treatment effects provided there is common support among the control population for the treated ones. Third, PSM does not answer the distributional effects of the variables, such as the percentage of AD patients that utilized more services.

This analysis found increased primary care utilization and costs for GP services by the AD patients compared to non-AD controls (Table 4). This is an important finding for three reasons. First, it supports the intuitive view that patients with AD have higher health care needs than their non-AD counterparts. Secondly, this study identifies that these increased needs are at least partially being met. Finally, it is revealed that the costs of meeting this need is substantial and has significant implications for health care budgets, given the aforementioned ageing population.

Our results were consistent with trends found in studies from the countries with similar socioeconomic conditions as Denmark.[18-21] A study carried out in the UK study found higher rates of primary care resource utilization in an AD cohort relative to general older adult control patients both prior and preceding the AD diagnosis.[18] Similarly, a Dutch study reported increased contact frequency by preclinical dementia to their GPs more than controls.[21] However, a few studies such as the ones conducted in the US[22] and France[23] showed no differences in primary care utilization among AD cases.

The number of additional GP services utilized by the AD patients compared to controls in the current study is consistent with the findings of the UK study that reported approximately 4 more consultations per 6 months on average.[18] Service utilization in the current study was, on average, around 20% more for AD patients as compared to the non-AD controls but the cost was only around 15% more on average (Table 4). This can be explained by the fact that the services utilized by AD patients are comparatively low cost compared to other services in Denmark, such as email and telephone consultations.

Literature regarding use of GP services by AD patients is scarce, particularly for Denmark. One study investigated costs of patients suffering from all dementias in Denmark, although GP services were not a specific focus.[24] The study found that demented cases utilized GP services less compared to the control group, although the overall cost for demented patients was significantly higher. Such costs were associated with a higher utilization of hospital services, rather than primary care. Kronborg et al. observed that the GP and medical consultation costs did not differ between patient groups. The mean costs found were almost half of the estimations in our study (Table 4). This might be due to the 15-year gap between studies during which the number of services, frequency of service utilization and costs might have actually increased in addition to annual inflation. However, such differences might have also arisen due to different methodologies adopted for estimations.

### Limitations and Strengths of the Study

A potential limitation of this study is the discrepancy between the real figure of people with dementia and the number of cases used in this study. Moreover, sensitivity analysis revealed possible hidden bias for unobservable covariates. Some studies have shown that the cost of care more than double between mild and severe dementia.[25-26] However, including stage-specific estimations was not possible due to lack of data.

The well-defined study population, the large sample obtained from a national database, controls matched for all observed covariates, based on propensity scores mimicking a RCT based on observed covariates and estimations based on completely matched data are important strengths of this study. Likewise, the use of GBM for estimating propensity score was efficient in dealing with uncertain functional forms of covariates and interactions among them, a major problem in model specification.[27] Resource use and cost data were obtained from national databases containing data about actual payments made, thus eliminating recall bias. This is evident from the fact that ATE and ATET calculated from the matched data almost coincided.

### Implications

This study has certain implications in terms of costs, research and methodology. The increased utilization and costs are likely to result in a higher total cost of primary care, an important implication for future health care resource allocation. This also has certain research implications. It was found that service utilization and costs among AD patients on average was over 20% and 15% higher, respectively. This might point to the fact that even though the AD patients utilize more services, the corresponding increase in costs might not be directly proportional. These findings imply that further research is required to confirm the pattern of service utilization and costs by the AD patients. Lastly, although PSM was successful in diminishing the imbalances in the covariate distribution among treatment groups, other efficient methods can be explored for better estimations. It has been found that Genetic Matching, a nonparametric matching that does not depend on knowing or estimating the propensity score, reduces the conditional bias as well as root mean squared error as compared to PSM leading to less biased estimates.[28-29] Genetic Matching brings about the reduction in biases even when the property of equal percent bias reduction (EBPR) does not hold. However, PSM performs poorly when such a property does not hold[29] and achieves better covariate balance than unadjusted analyses of the RCT data.[28]

## CONCLUSIONS

The study concludes that people with AD utilize more services as well as incur higher GP related costs. PSM aided in reducing the imbalance in observed covariates to large extent, provided a balanced matched data and improved the estimations. However, sensitivity analysis showed there may be some potential hidden bias. Inclusion of important health related covariates like quality of life and functional status to would be necessary to address the hidden bias.

## CONFLICT OF INTEREST DECLARATION

The authors declare that they have no conflict of interest. Funds provided by Pfizer Denmark A/S were partly used to fund this study.

## References

1 Maalouf M, Ringman J, Shi J: An update on the diagnosis and management of dementing conditions. *Rev Neurol Dis* 2010;8:e68-87.

2 Mathers CD, Loncar D: Projections of global mortality and burden of disease from 2002 to 2030. *PLoS Med.* 2006;3:e442.

3 Danish Dementia Research Center: Prevalence of Dementia in Denmark. 2014.

4 National Board of Health MaHTA: Diagnostic Evaluation and Treatment of Dementia-a health technology assessment. 2008. http://sundhedsstyrelsen.dk/~/media/70A72DEBDFE14DFBA39981CC75556A64.ashx.

5 Austin PC: An introduction to propensity score methods for reducing the effects of confounding in observational studies. *Multivariate Behavioral Res.* 2011;46:399-424.

6 Lynge E, Sandegaard JL, Rebolj M: The Danish national patient register. *Scandinav J Public Health.* 2011;39:30-3.

7 Kildemoes HW, Sørensen HT, Hallas J: The Danish national prescription registry. *Scandinav J Public Health.* 2011;39:38-41.

[8] Bauer K, Schwarzkopf L, Graessel E, Holle R: A claims data-based comparison of comorbidity in individuals with and without dementia. *BMC Geriatrics* 2014;14:10.

[9] Guo S, Fraser M: Propensity score analysis: Statistical methods and applications. Thousand Oaks, California: *SAGE*, 2009.

[10] Ho DE, Imai K, King G, Stuart E: MatchIt: nonparametric preprocessing for parametric causal inference. *J Stat Software* 2011;42(8):1-28.

[11] Rosenbaum PR, Rubin DB: The central role of the propensity score in observational studies for causal effects. *Biometrika* 1983;70:41-55.

[12] Rosenbaum PR: Hodges-Lehmann point estimates of treatment effect in observational studies. *J Am Stat Assoc* 1993;88: 1250-3.

[13] Normand S-LT, Landrum MB, Guadagnoli E, et al: Validating recommendations for coronary angiography following acute myocardial infarction in the elderly: A matched analysis using propensity scores. *J Clin Epidemiol* 2001;54:387-98.

[14] Hill JL, Reiter JP, Zanutto EL: A comparison of experimental and observational data analyses. In: Applied Bayesian modeling and causal inference from incomplete-data perspectives: An essential journey with Donald Rubin's statistical family. *Wiley*, 2004:49-60.

[15] Finkelstein MO, Levin B: Statistics for lawyers. *Springer*, 2001.

[16] Rubin DB: Estimating causal effects from large data sets using propensity scores. *Ann Intern Med* 1997;127:757-63.

[17] Bryson A, Dorsett R, Purdon S: The use of propensity score matching in the evaluation of active labour market policies. 2002.

[18] Chen L, Reed C, Happich M, Nyhuis A, Lenox-Smith A: Health care resource utilisation in primary care prior to and after a diagnosis of Alzheimer's disease: a retrospective, matched case-control study in the United Kingdom. *BMC Geriatrics* 2014;14:76.

[19] Albert SM, Glied S, Andrews H, Stern Y, Mayeux R: Primary care expenditures before the onset of Alzheimer's disease. *Neurology*. 2002;59:573-8.

[20] Eisele M, van den Bussche H, Koller D, et al: Utilization patterns of ambulatory medical care before and after the diagnosis of dementia in Germany – Results of a case-control study. *Dementia Geriatric Cognitive Disord* 2010;29:475-83.

[21] Ramakers IHGB, Visser PJ, Aalten P, et al: Symptoms of preclinical dementia in general practice up to five years before dementia diagnosis. *Dementia Geriatric Cognitive Disorders*. 2007; 24: 300-6.

[22] Leibson C, Owens T, O'Brien P, et al: Use of physician and acute care services by persons with and without Alzheimer's disease: a population-based comparison. *J Am Geriatr Soc* 1999;47:864-9.

[23] Helmer C, Pérès K, Pariente A, et al: Primary and secondary care consultations in elderly demented individuals in France. *Dementia Geriatr Cognitive Disord* 2008;26:407-15.

[24] Kronborg Andersen C, Søgaard J, Hansen E, et al: The cost of dementia in Denmark: the Odense Study. *Dementia Geriatr Cognitive Disord* 1999;10:295-304.

[25] Quentin W, Riedel-Heller SG, Luppa M, Rudolph A, König HH: Cost-of-illness studies of dementia: a systematic review focusing on stage dependency of costs. *Acta Psychiatrica Scandinavica* 2010;121:243-59.

[26] Leicht H, Heinrich S, Heider D, et al: Net costs of dementia by disease stage. *Acta Psychiatrica Scandinavica* 2011;124:384-95.

[27] McCaffrey DF, Ridgeway G, Morral AR: Propensity score estimation with boosted regression for evaluating causal effects in observational studies. *Psychological Methods* 2004;9:403.

[28] Sekhon JS, Grieve RD: A matching method for improving covariate balance in cost-effectiveness analyses. *Health Econ* 2012;21:695-714.

[29] Diamond A, Sekhon JS: Genetic matching for estimating causal effects: A general multivariate matching method for achieving balance in observational studies. *Rev Econ Stat* 2013;95:932-45.
